# How to Implement Clinical Practice Guidelines in Iran

**DOI:** 10.5812/ircmj.9702

**Published:** 2013-11-05

**Authors:** Reza Majdzadeh, Zahra Baradaran Seyed

**Affiliations:** 1 School of Public Health, Tehran University of Medical Sciences, Tehran, IR Iran

**Keywords:** Practice Guidelines, Health Plan Implementation, Early Intervention

## Abstract

**Background:**

Evidence-based medicine would come to the result by evidence-based implementation. Clinical Practice Guidelines (CPG) as one of the evidence-based knowledge products requires appropriate interventions after being produced to be applied.

**Objectives:**

The aim of this qualitative study was to identify the strategies for application of CPGs produced in Iran.

**Materials and Methods:**

The purposive snowball sampling was performed and it continued until reaching the theoretical saturation. In-depth semistructured individual interviews and Focus Group Discussion (FGD) were performed aiming at triangulation. The thematic framework approach was used for the analysis.

**Results:**

Twelve interviews were conducted with three health system policy makers and decision makers, four experienced in the production or adaptation of clinical practices, and five experts in evidence-based medicine development and education. In addition, 11 policy makers, managers, and decision makers of the health system took part in FGD. The proposed interventions were classified in the following themes: Health professionals-oriented, Financial, Organizational, Regulatory, and Multifaceted interventions.

**Conclusions:**

Along with adaptation and development process of CPGs, their utilization should also be planned; otherwise spent time and money would be in vain. Certainly, imposing above-mentioned interventions with the ultimate goal of sustainable behavior change in health system service providers is beyond the capacity of specific groups or few academic centers. It requires the participation of all practitioners under the monitoring and support of the Ministry of Health and Medical Education. Deployment of the family physician plan and referral system is an opportunity which must be considered a trophy.

## 1. Background

Related activities to Evidence-Based Medicine (EBM) development and education were initiated for more than a decade in Iran. However, there is a long distance to EBM (1). Considering evidence-based knowledge products implementation, which facilitates EBM is much emphasized (1, 2). Clinical Practice Guidelines (CPGs) as one of the knowledge products with the aim of improving the clinical service providing, not only plays a role as a professional aid for practitioners and patients, but also as a tool for insurances with the purpose of cost control and for governments with the purpose of policy making or prioritizing the health care. For policy makers, cost-effectiveness issue is at least as important as the quality of health care and these tools are useful for avoiding unnecessary treatments and additional costs (3). Despite the regulation for producing such products, few CPGs have been produced or adapted in the country, and their utilization in the country's health system has not been observed yet (4). In a conducted study for the investigation of awareness and attitude of Tehran's physicians toward clinical guidelines, it was found that only 31.8% of physicians were familiar with the guidelines. This familiarity included having a manual of in-house or even foreign clinical guidelines, and it did not include adherence to CPG (5). In addition, findings of a qualitative study investigating the barriers for using clinical guidelines in Iran, suggested that barriers for the establishment of development and implementation of the CPG system at six levels include: practice environment, evidence-based health care system, individual professional, politician and political context, innovation (CPG), and patients (4). In fact, lack of evidence-based health care system as well as absence of suitable political support at the macro level in Iran as a developing country has challenged the establishment of CPG development and implementation system (4, 6). However, implementation of the family physician plan and referral system is an opportunity, so that development and adaptation of evidence-based clinical guidelines are followed systematically and seriously (6). In this way, it is necessary to apply appropriate interventions to ensure their utilization after development and adaptation of evidence-based clinical guidelines suitable for local conditions. The present study aimed to address effective strategies and interventions to overcome barriers for CPG implementation in Iran.

## 2. Objectives

This study, including oral consent of participants, was approved by the institutional review board of Tehran University of Medical Sciences, which follows the Helsinki declaration. Oral consent was obtained since the study did not contain any procedure or question that could harm study subjects. The study subject and objectives were introduced for all participants at the beginning of interviews, and then oral consent was obtained. A colleague witnessed the oral consent of all participants in which we had more than one interviewer and for cases of having single facilitator the consent was recorded. The study subjects had the right to withdraw from the study whenever they wanted, and the recording was stopped in any circumstances they asked. Participants were anonymous in the paper and also in the report of the study.

The research team had considered arrangements for decreasing bias and reflexivity due to their own point of views and preferences; the interviewer (ZBS) had participated in training programs on methodology, analysis and interpretation of qualitative studies, and also the team extensively reviewed the literatures on clinical guideline development in other countries.

All data were analyzed including transcribed interviews, audio-recorded interviews, as well as the field notes by the interviewer. The analysis performed by thematic framework approach. After listening and frequent reading of interviews and literature review aimed at familiarization, general themes were extracted and the thematic framework was specified (7, 8). Then, Indexing and Charting were performed using MAXQDA® 10 software which is a “computer assisted qualitative data analysis” (CAQDAS) package (9). Primary themes and subthemes were peer-examined and subsequently indexing was performed by the final, approved, framework. Then, charting in two axis of vertical and horizontal was used which resulted in analysis of themes and selected content of interviews according to the different study groups. At last, mapping and interpretation were performed, and the final result of the study and its framework were shared with stakeholders for member checking.

## 3. Materials and Methods

This research was a qualitative study with a purpose of applied policy research, therefore, the thematic framework approach was used (9). The sampling was performed by identifying stakeholders in the maximum variation. The inclusion criteria for purposive snowball sampling were familiarity with CPGs and former measures on implementation of EBM in Iran. Therefore, Interviews were conducted with health system policy makers and decision makers, experienced in production or adaptation of clinical practices and experts in evidence-based medicine development and education. The interviews continued until reaching the theoretical saturation (10). The primary interview guideline was prepared, and further ad-hoc questions were used according to the context.

In-depth semistructured individual interviews and focus group discussion (FGD) were performed aiming at triangulation. There were not any exclusion criteria.

## 4. Results

Twelve interviews were conducted with three health system policy makers and decision makers, four experienced in production or adaptation of clinical practices and five experts in evidence-based medicine development and education. In addition, 11 policy makers, managers and decision makers of the health system took part in FGD. The proposed interventions were classified in the following themes: Health professionals-oriented, Financial, Organizational, Regulatory, and Multifaceted interventions.

Selected parts mentioned by the interviewees in policy and decision maker group (in-depth interview and FGD) were denoted as code 1, by experts in guidelines development and adaptation were denoted as code 2, and those by experts in evidence-based medicine development and education were denoted by Code 3 as italic.

### 4.1. Health Professionals-Oriented Interventions

Service provider-based interventions accounted for a considerable share for the number of interventions. The highest share in this regard was for education and entrance in educational curriculum:

*(1) resident should learn how to use a guideline in bedside at the hospital, that is, board examination should follow the local guideline*

*(2) we should hold an educational course for health service network physicians which are to use the guidelines… and we should teach them what evidence it is! such concepts should be clarified… it should be clarified that output of evidence-based medicine is the clinical practice guideline. It may eliminate most of these barriers.*

Although there were participants indicating that expectations from education effectiveness should be adjusted:

*(1) if I were asked: "to which extent change occurs by education", I am sure that it would not be over 5 to 10 percent in my opinion, education is much beyond that we put guidelines beside the textbooks.*

Regarding distribution and dissemination, some hints were mentioned:

*(1) for example, I offer a publish saying "Medical Council, You should publish any guidelines.., "various associations! One of your tasks should be publishing guidelines", "Ministry of Health! You should use these in your database", "related universities! You should use these in your database"*

*(1) for example, if my job requires designing a flowchart or algorithm to cover all of my work, I should have performed it already so that physician can perform his or her job easily. If I was unable to do so, I should provide basic recommendations of guidelines in an A4 paper so that he really knows what to do and what decision should be made.*

Another intervention with lower weight was outreach visiting or taking the help of intellectual leaders and emphasis was put on it being purposeful:

*(2) heads of the departments should be talked and convinced. The behavior you show is not important for a hospital, but all should do one thing it should not be in such a way that a similar disease is treated by three different methods in a hospital.*

*(1) well, we should do something that opinion leaders believe in the guideline, this needs an extensive culture making.*

Feedbacks were also mentioned:

*(2) how they know they are misdoing? Each medicine prescribed for patient and any protocol used for the patient should be checked already in the pharmacy. Then, the Health Insurance exactly monitors. As soon as they recognize improper use or using self-preferred drugs, what would they do? Send feedback.*

### 4.2. Financial Interventions

Financial interventions were also mentioned; of course, emphasis was put on preventive financial interventions:

*(1) we must say: hospital! If you do not follow this guideline, 20% of your total income would be cut.*

Imposing financial incentive interventions was considered as challenging:

*(2) I do not think the financial incentive well developing criteria is very difficult and I think it would become vain, that is, people would think in another way, they would say certainly there was an agreement with the Aspirin company that we should do this.*

Macro outlook was based on the necessity for the change in the payment system:

*(3) in my opinion, the more ideal condition, the more health system resources are specified, contribution of different people is formed. When it is created, it can correct monitoring mechanisms very well. For example, if we have a system in which people contribute in health system costs in proportion to their income level, the health system can provide health care without the need for direct payment, or with low costs like countries with universal insurances, then more power is gained for better monitoring of performance of clinical specialists and various groups now most in-patient and out-patient services are not under insurance coverage. If insurance coverage is high, insurance firms can control these behaviors by some mechanisms, but until then it is somehow difficult to control. I argue that if we are the idealist, the better is that service delivery basis should be corrected in such a way that all clinical actions be performed under surveillance of the health system.*

*(3) if we eliminate direct financial relation between physician and patient and monitoring bodies are put as the filter between them, it provides a good mechanism to control the clinical behaviors. In this way, since payments and some other things become dependent to adherence of people to specific paths, protocols and guidelines, implementation can be performed easier. Supposed that our insurance coverage is much higher, this condition occurs.*

### 4.3. Organizational Interventions

In organization intervention section, although some strategies were proposed, there were not considerable variety and frequency, and it included structural and staff-oriented interventions:

*(1) necessary information infrastructures should be developed in service delivery centers*

*(2) for example, one person should be located in the drugstore to check all the chemotherapy drugs that have been prescribed and to identify what have been prescribed correctly; he or she should have some valid pattern and guideline, so that he or she can easily recognize, say drugs A and B, have no indications at least much financial waste can be prevented, but it is the case provided that experienced people are located in insurance.*

### 4.4. Regulatory Interventions

Participants insisted on legal and regulatory interventions. In this regard, the prohibition of intervention by pharmacological companies' visitors with legal pressures was discussed:

*(1) it is enough that you simply mention to one of these companies and say: "you are not allowed to say to my physician to prescribe this drug". It works. That is, one of such major companies should be fined so that others know the circumstances.*

In addition, interventions in the development of clinical guidelines were also discussed:

*(1) it is just enough that the minister holds a meeting today and summons involved people in clinical guidelines and asks them in this regard if there is also a robust national team monitoring implementation, in my opinion, it would progress easily.*

*(2) perhaps, it needs a guarantee action and we shall say if an academic department does not produce one guideline annually, its accreditation would be revoked and it would be turned into a nonacademic group*

On the other hand, interventions were also recommended in the implementation of guidelines:

*(1) implementation of guidelines should be tied to existence and nonexistence of the providers if just this one intervention occurs; there would be much difference*

*(3) above all, the law should support me. The national guideline is like a book. Even if someone is treated and died due to it, it does not matter. Because, it has become accredited and approved and it should be followed. It is regarded as a "must", not "I want" or "I do not want"*

However, some disregard legal obligation:

*(1) when you write a guideline, you should not force the physician. It is possible that one patient would not be treated by this method*

*(2) The guideline is regarded as an aid for you, but you are not obliged to adhere. The guideline says: "this method is the best", but I as a physician, even in the USA, I am free to change my approach according to the case.*

### 4.5. Multifaceted Interventions

Finally, multifaceted and simultaneous interventions were recommended for effectiveness:

*(1)… it needs insurance, law, peer pressure and so on. Guideline production should be one of the scores for upgrading… suppose that insurance system, reward and punishment system, and, say, system for prescriptions reviews, all must be consistent with guidelines. If this is going to happen.*

*(2) it should have a scientific and structured framework. It should be localized based on specific characteristics of the guideline. It should be trained as retraining. As an obligation, they should be said if they act in such a way other than the guideline and the patient was troubled, they should be held accountable to the Medical Council.*

## 5. Discussion

Employing CPG has become more important after the establishment of the referral system and the family physician plan (6). As mentioned, CPGs can be served as a tool for promoting evidence-based service delivery and external control, insurance firms, governments, and public organizations (3). In the Europe, the cost of development of CPGs is between 10,000-200,000 Euro. Of course, the cost of distribution and implementation of these guidelines should also be added. The necessity of considering strategies is more important to make implementation of CPGs possible for the practitioners (11, 12). It is necessary to select a combination of interventions with approved cost-effectiveness and assessed value. To this end, attracting upstream budget and support are essential and organizational aspects stimulate and encourage the usage of guidelines should be considered (11-14).

The analysis of present qualitative study was a thematic framework with a purpose of applied policy research. Principally its approach is deductive and really we did not prospect to find a novel theory. Despite authorities did not implement in-house adapted CPGs in Iran yet, EBM and CPG implementation are not new phenomena internationally. Therefore, we conducted this thematic framework qualitative study to find a useful framework to help policy and decision makers to implement CPGs in Iran context. As it was mentioned in the result section, interventions recommended by interviewees included health professionals-oriented, financial, organization, regulation, and multifaceted interventions.

The mentioned themes were generally consistent with recognized international strategies; however, application of each strategy had less variety (7, 8, 13). The mentioned themes were discussed in details as follows.

### 5.1. Health Professionals-Oriented Interventions 

For interviewees, the highest frequency in service delivery-based interventions was for education. One way for training content of clinical guidelines is embedding them in the educational curriculum of family-physician or residency (15). However, mere focus on this section should be considered carefully, it has been known that despite a long history of evidence-based medicine education in the country, there has not been the required change in behavior of clinicians toward evidence-based service delivery (1).

One of the service delivery-based interventions is the related actions for the distribution and dissemination of clinical guidelines (7, 11). One important point is repetition in a specific period of time. The other point is considering approaches, which are published as written and text. Most target groups never read the material or read it incompletely or read only those parts, which are matched to their preferences and tendencies (13). Knowing the main sources used by the target group is useful in selecting the suitable strategies. Sources of information for physicians are different depending on the membership to target groups. Scientific journals are introduced as the primary information sources mostly by the members of the scientific and professional societies, younger physicians and clinicians involved in education, while pharmaceutical companies' vendors are information sources mostly for those groups working independently and they are not the member of any associations (16). Results of a cross-sectional study investigating the situation of knowledge promotion in 319 general practitioners toward diabetes in Tehran showed that the most-used sources were local journals, and CPGs had no place in this regard (17).

Another recommendation was audit and feedback, though it accounted for a small part. Effectiveness of feedbacks varies from one study to another, and it is influenced by such factors as motivation of the recipient, timing and frequency of sending feedback, information type, such as resources and recommendations and so on (18-21). Comparative feedbacks containing peer information can be effective in the use of feedback data. Feedbacks may be about the cost, treatment outcomes, number of drugs or the type of prescription and they are generally retrospective and are reached by target group after the event (18-21). However, it is not clear that how long after the event change, you should continue to send feedback, but as a usual rule, the tendency of the provider is to return to his or her previous practice, and thus to continue the feedback sending would be required for a long-time (22).

Other interventions discussed in this section but not mentioned by interviewees can be holding a conference and meeting and so on aiming at education, patient-mediated interventions, reminders and finally peer reviews (7, 8, 13).

### 5.2. Financial Interventions 

Financial interventions in the current study referred to disciplinary and incentive interventions. Furthermore, the need to shift in payment for cost of care by patients was emphasized. Generally, financial interventions are focused on the service provider or patients. At the service provider level, such payment methods as a fee for services, per capitation and provider salaried service could be mentioned. In addition, direct and indirect financial incentives and rewards, financial assistance or benefits paid directly or indirectly, direct or indirect financial penalties, and increased or withdrawal of products available and affordable can also be mentioned. On the other hand, financial interventions are directed to the patient, including insurance premiums, copayments by patients, user charges, patient incentives, grants and privileges, fines, and so on (9, 13). As mentioned by the interviewees, until the payment system is not corrected, the adherence to the clinical guidelines cannot be expected. Full implementation of the referral system and disconnecting financial relationship between the patient and the therapist provide setting for implantation of interventions. In this case, insurances are paid only when diagnosis and treatment process have been conducted according to the clinical guidelines (6).

### 5.3. Organizational Interventions

Generally, organizational interventions are classified into three classes including structural, staff-oriented, and patient-oriented interventions (7). In the present study, only structural and staff- oriented interventions were mentioned by the interviewees.

Structural interventions are as follows: change in structure or location of service delivery, telemedicine, medical recording system changed from paper forms to computer settings, change in setting and maintenance or deletion of information such as patient follow-up system, changes in the scope and range of services, changes in the nature or organization of quality management mechanisms, change in ownership or affiliation status of hospitals or other equipment (7, 13, 23). In staff-oriented interventions, revision of professional roles, a multidisciplinary team, integrated care that may include coordination of patient's received care across the unit boundaries (seamless care) can be named. Changing the number or adjusting staff, interventions to improve provider satisfaction with working conditions or his or her resources or psychological rewards and morale promotion are among other staff-oriented interventions (7, 13).

However, patient-oriented interventions were not mentioned as effective interventions in the application of CPGs by interviewees in this study, the results of interventions based on or mediated by the patients showed that they are not necessarily consistent with the improvement of health service delivery, though some cases led to positive evaluation and more satisfaction in patients (23), Moreover, when patients prefer special treatment, which is not based on a scientific evidence-based method, patient participation may lead to reduced adherence to the guidelines (24).

### 5.4. Regulatory Interventions

Interventions performed by regulation and by laws as aiming at change in service or cost, which may overlap with financial and organizational interventions, may include different types of interventions. The types include changes in medical liability, claims and litigation case management, accreditation and licensing and permitting the practice, and etc. (7, 13). Despite emphasis on the obligation for adherence to clinical guidelines, clinical guidelines have some limitations. In fact, these tools provide supposed standards for the patients and specifically do not consider the special condition of individuals and patients with their preferences. Thus, obligation for service providers to implement recommendations necessarily and accurately would not follow quite satisfactory outcomes (25). However, in the current condition of our country and in line with the implementation of a referral system and public insurance coverage, the only way for controlling the costs could be development and adaptation of the clinical guidelines which are useful for monitoring diagnosis and treatment processes. In this way, a mechanism is needed for reviewing the exceptions by expert working teams, so that no interruption occurs in insurance coverage enjoyment, if CPG is not adhered.

### 5.5. Multifaceted Interventions

Although one or more specific combinations of interventions cannot be referred as a priority over other interventions, evidences suggest that provider-based interventions such as education, feedback and reminders are more effective than organizational or financial interventions or patient-oriented ones (13, 26). Effectiveness of multifaceted intervention depends on the effectiveness of all involved interventions as well as their interaction which may lead to an increase or decrease in overall effect. Increasing the number of interventions does not necessarily mean more effectiveness and it would impose the high costs (20, 27, 28). Generally, the selection of individual or multifaceted interventions depends on the topic, target group, change the context and the problems. If there is a budget limitation, strategy selection would depend on the effectiveness; the important point is that different aspects of barriers are covered in types of interventions; if education can overcome the lack of knowledge and feedback, the combination of both issues can eliminate two barriers to behavior change simultaneously (11, 13).

Overall, strategies focused on reduction of time burden and task complexity compared to interventions in education and incentives, which have limited effects, can improve the performance. Essentially, the strategies for implementing guidelines should be designed for reducing the barriers in working conditions with high pressure and time burden such as primary care condition, but intervention strategies would have more effectiveness at system and organization levels compared to individual level of the service provider (29).

The barriers and appropriate solution could be changed, depending on the level and progress of CPG development in the country, which could be considered as the study weakness. This issue must be considered in generalizability of the present findings. The barriers and solution could be changed upon the focus of health authorities and or commitment of insurance organization(s) on using CPGs differed.

The important strength of study is diversity, which resulted from maximum variation sampling. By this, the study appropriately elaborated the barriers in the country and the proposed context based interventions for development and implementation of CPG.

Finally, along with the development and adaptation of CPGs, their implementation should also be planned; otherwise spent time and money would be in vain. Imposing health professionals-oriented, financial, organizational, regulatory and multifaceted interventions with the ultimate goal of sustained behavior change in health system providers is certainly beyond the capacity of specific groups or few academic centers. It requires the participation of all stakeholders under the monitoring and support of the Ministry of Health and Medical Education. Deployment of the family-physician plan and referral system is an opportunity which must be considered for eliminating one of the major barriers for implementing CPGs, that is, lack of macro level political support and lack of an evidence-based caring system.
